# No Evidence for Activated Autophagy in Left Ventricular Myocardium at Early Reperfusion with Protection by Remote Ischemic Preconditioning in Patients Undergoing Coronary Artery Bypass Grafting

**DOI:** 10.1371/journal.pone.0096567

**Published:** 2014-05-05

**Authors:** Nilgün Gedik, Matthias Thielmann, Eva Kottenberg, Jürgen Peters, Heinz Jakob, Gerd Heusch, Petra Kleinbongard

**Affiliations:** 1 Institut für Pathophysiologie, Universitätsklinikum Essen, Universität Duisburg-Essen, Essen, Germany; 2 Klinik für Thorax- und Kardiovaskuläre Chirurgie, Universitätsklinikum Essen, Universität Duisburg-Essen, Essen, Germany; 3 Klinik für Anästhesiologie und Intensivmedizin, Universitätsklinikum Essen, Universität Duisburg-Essen, Essen, Germany; KRH Robert Koch Klinikum Gehrden, Germany

## Abstract

**Objective:**

Remote ischemic preconditioning (RIPC) by repeated brief limb ischemia/reperfusion reduces myocardial injury in patients undergoing coronary artery bypass grafting (CABG). Activation of signal transducer and activator of transcription 5 (STAT5) in left ventricular (LV) myocardium at early reperfusion is associated with such protection. Autophagy, i.e., removal of dysfunctional cellular components through lysosomes, has been proposed as one mechanism of cardioprotection. Therefore, we analyzed whether or not the protection by RIPC is associated with activated autophagy.

**Methods:**

CABG patients were randomized to undergo RIPC (3×5 min blood pressure cuff inflation/5 min deflation) or placebo (cuff deflated) before skin incision (n = 10/10). Transmural myocardial biopsies were taken from the LV before cardioplegia (baseline) and at early (5–10 min) reperfusion. RIPC-induced protection was reflected by decreased serum troponin I concentration area under the curve (194±17 versus 709±129 ng/ml × 72 h, p = 0.002). Western blotting for beclin-1-phosphorylation and protein expression of autophagy-related gene 5–12 (ATG5-12) complex, light chain 3 (LC3), parkin, and p62 was performed. STAT3-, STAT5- and extracellular signal-regulated protein kinase 1/2 (ERK1/2)-phosphorylation was used as positive control to confirm signal activation by ischemia/reperfusion.

**Results:**

Signals of all analyzed autophagy proteins did not differ between baseline and early reperfusion and not between RIPC and placebo. STAT5-phosphorylation was greater at early reperfusion only with RIPC (2.2-fold, p = 0.02). STAT3- and ERK1/2-phosphorylation were greater at early reperfusion with placebo and RIPC (≥2.7-fold versus baseline, p≤0.05).

**Conclusion:**

Protection through RIPC in patients undergoing CABG surgery does not appear to be associated with enhanced autophagy in LV myocardium at early reperfusion.

## Introduction

Remote ischemic preconditioning (RIPC) is an attractive strategy to attenuate perioperative myocardial damage resulting from ischemia/reperfusion injury [1]–[3] and to improve the prognosis of patients undergoing coronary artery bypass grafting (CABG) [4]. However, the transfer of the protective signal from the ischemic/reperfused periphery to the heart and the protective signal cascade within the myocardium remain largely unknown [5]. Recently, we found the activation of the signal transducer and activator of transcription 5 (STAT5) in left ventricular (LV) myocardium at early reperfusion to be associated with protection by RIPC in patients undergoing CABG [6].

Autophagy is a process whereby double-membrane vesicles (autophagosomes) remove dysfunctional cellular components through fusion with lysosomes; the autophagosome content is then degraded and recycled [7], [8]. In mouse hearts *in vivo* [9] and in isolated rabbit hearts [10] autophagy was induced by ischemia and further enhanced by reperfusion. The activation of autophagy is reflected by increases in the abundance of key proteins of the autophagy-related pathways: beclin-1, light chain 3 (LC3), autophagy-related gene 5-12 complex (ATG5-12), and p62 [11]–[14]. With progress of autophagy, the autophagic proteins themselves will be degraded and thus their abundance decreased [11]–[13]. In particular, the time window for the increase of p62 is short [14], and activation of autophagy is often associated with a decrease of p62 [12]. In biopsies from the right atrial appendage of patients undergoing CABG or valve surgery, the expression of ATG5-12, beclin-1, LC3-I, LC3-II, and p62 was in fact decreased during reperfusion [13].

Autophagy activation has been proposed as one mechanism of cardioprotection [15]. In isolated rat hearts, protection by ischemic preconditioning, i.e., brief episodes of coronary artery occlusion/reperfusion, was associated with enhanced myocardial expression of LC3-II, beclin-1 [16], and p62 [14] as well as with enhanced expression of parkin in the mitochondrial fraction [17]. Parkin is a requisite for autophagic removal of mitochondria [17]. Mitochondria are potential end-effectors of cardioprotection and decisive for cardiomyocyte survival at early reperfusion [18]. Protection by autophagy activation is proposed to be related to the elimination of dysfunctional and damaged mitochondria [17], [19].

The involvement of autophagy activation in myocardial protection by RIPC, notably in the human heart, has not yet been addressed. Accordingly, we analyzed established autophagy markers by Western immunoblotting in LV myocardial biopsies before ischemic cardioplegic arrest and during early reperfusion in patients undergoing elective CABG with and without RIPC.

## Material and Methods

### Ethics Statement

With approval of the local ethics committee (Germany: Institutional Review Board, University of Duisburg-Essen) and patients' written informed consent we analyzed LV biopsies from patients having undergone elective isolated first-time CABG between August 2010 and May 2012 who were enrolled in a randomized, prospective, double-blind, placebo-controlled study without and with RIPC (ClinicalTrials.gov NCT01406678).

### Study procedure

The inclusion and exclusion criteria for the trial have been reported [4]. Samples were available from 20 patients (10 RIPC and 10 placebo, all undergoing surgery with isoflurane anesthesia), of which 3 in each group had been analyzed before [6].

General anesthesia was induced with sufentanil (1 µg/kg), etomidate (0.3 mg/kg) and rocuronium (0.6 mg/kg) and maintained with isoflurane (0.6–1.0% end-tidal). The RIPC protocol consisted of 3 cycles of 5 min left upper arm ischemia/5 min reperfusion and was compared to placebo (cuff left deflated for 30 min). Surgical revascularization was performed in all patients using median sternotomy, mild systemic hypothermia (>32°C) and antegrade cold crystalloid Bretschneider (Köhler Chemie GmbH, Bensheim, Germany) cardioplegia with additional topical cooling and single aortic cross-clamping for all distal anastomoses.

Venous blood samples were drawn from each patient preoperatively on the day before surgery and postoperatively at 1, 6, 12, 24, 48, and 72 h and analyzed for serum troponin I concentration (cTnI) by a specific two-side immunoassay (Dimension Flex, Dade Behring GmbH, Marburg, Germany) with a detection range of 0.04–40 ng/ml. The reference interval was zero to 0.05 ng/ml. Values above 0.1 ng/ml were considered as abnormal. The area under the curve (AUC) for serum cTnI was calculated according to the trapezoidal rule. Missing values were replaced by linear inter- and extrapolation.

### Myocardial biopsies and Western Blot analysis

Transmural myocardial biopsies of 2–5 mg were taken at baseline before initiation of cardiopulmonary bypass (CPB) and at 5–10 min reperfusion following aortic unclamping from the respective LV perfusion territory undergoing revascularization using a Tru-Cut R biopsy needle (Cardinal Health, Dublin, OH, USA). Biopsies were quickly frozen in liquid nitrogen and stored at −80 °C until subsequent Western Blot analysis. Frozen myocardial biopsies were homogenized in 1× RIPA buffer (Cell Signaling, Danvers, MA, USA), supplemented with 1× Complete Protease Inhibitor Cocktail and 1× PhosSTOP Phosphatase Inhibitor Cocktail (Roche, Basel, Switzerland) using a mixer mill (Retsch, MM301, Haan, Germany). After centrifugation at 14.000 g for 10 min at 4 °C and recovery of supernatants, protein concentrations were determined using the detergent compatible protein assay (Biorad, Hercules, CA, USA). Protein aliquots of 10 µg were electrophoretically separated on 10% or 12% SDS-PAGE and transferred to membranes. After blocking with 5% non-fat dry milk, the membranes were incubated with primary antibodies against the following key proteins of autophagy [11], [12], [17]: ATG5-12 complex (APG5, Santa Cruz Biotechnology, Dallas, TX, USA), phospho-beclin-1 (Abgent, San Diego, CA, USA), beclin-1, LC3 (LC3B, Cell Signaling, Danvers, MA, USA), parkin (Santa Cruz Biotechnology, Dallas, TX, USA) and p62 (PM045; MBL International, Woburn, MA, USA). Antibodies against phospho-STAT5 and STAT5 (Cell Signaling, Danvers, MA, USA) were used to confirm the association of STAT5 activation with cardioprotection by RIPC, and antibodies against phospho-STAT3, STAT3, phospho-extracellular signal-regulated protein kinase 1/2 (ERK1/2) and ERK1/2 (Cell Signaling, Danvers, MA, USA) were used to confirm signal activation by ischemia/reperfusion [6]. After incubation with the respective horseradish peroxidase conjugated secondary antibody (Cell Signaling, Danvers, MA, USA) immunoreactive signals were detected by chemiluminescence (SuperSignal West Femto Maximum Sensitivity Substrate, Pierce, Rockford, Il, USA) with a ChemoCam Imager (Intas Science Imaging Instruments GmbH, Göttingen, Germany) and quantified with LabImage 1D software (Kapelan Bio-Imaging GmbH, Leipzig Germany).

Immunoreactivities of the autophagy-related proteins ATG5-12, beclin-1, parkin and p62 were normalized to glyceraldehyde 3-phosphate dehydrogenase (GAPDH). For p62, the quantification was done with the upper of two apparent bands [13]. For the quantification of LC3 it is recommended to normalize LC3-II to a housekeeping protein or to LC3-I; therefore we have normalized LC3-II to both GAPDH and LC3-I [12]. Immunoreactivities of phosphoproteins were normalized to their respective total proteins. Ponceau staining was used to normalize for equal protein loading of the gels. To exclude a potential decrease of GAPDH during ischemia/reperfusion, GAPDH was normalized to Ponceau staining. The GAPDH/Ponceau-ratios for all gels were not different between RIPC and placebo and between baseline and early reperfusion, respectively (Figures S1, S2, S3, S4). Samples were compared between baseline and early reperfusion within the placebo and the RIPC group separately, and both, baseline and reperfusion samples were also compared between placebo and RIPC on different blots.

### Statistical analysis

Data are expressed as mean ± standard error of the mean (SEM). Serum cTnI were analyzed by 2-way (group, time) ANOVA for repeated measures. The AUC for the serum cTnI over 72 h was compared by unpaired Student t-test. Immunoreactivities on the same blot were compared by paired (within RIPC and placebo, respectively) or unpaired (between RIPC and placebo) Student t-tests. Differences were considered significant at the level of p<0.05.

## Results

**Figure 1:**
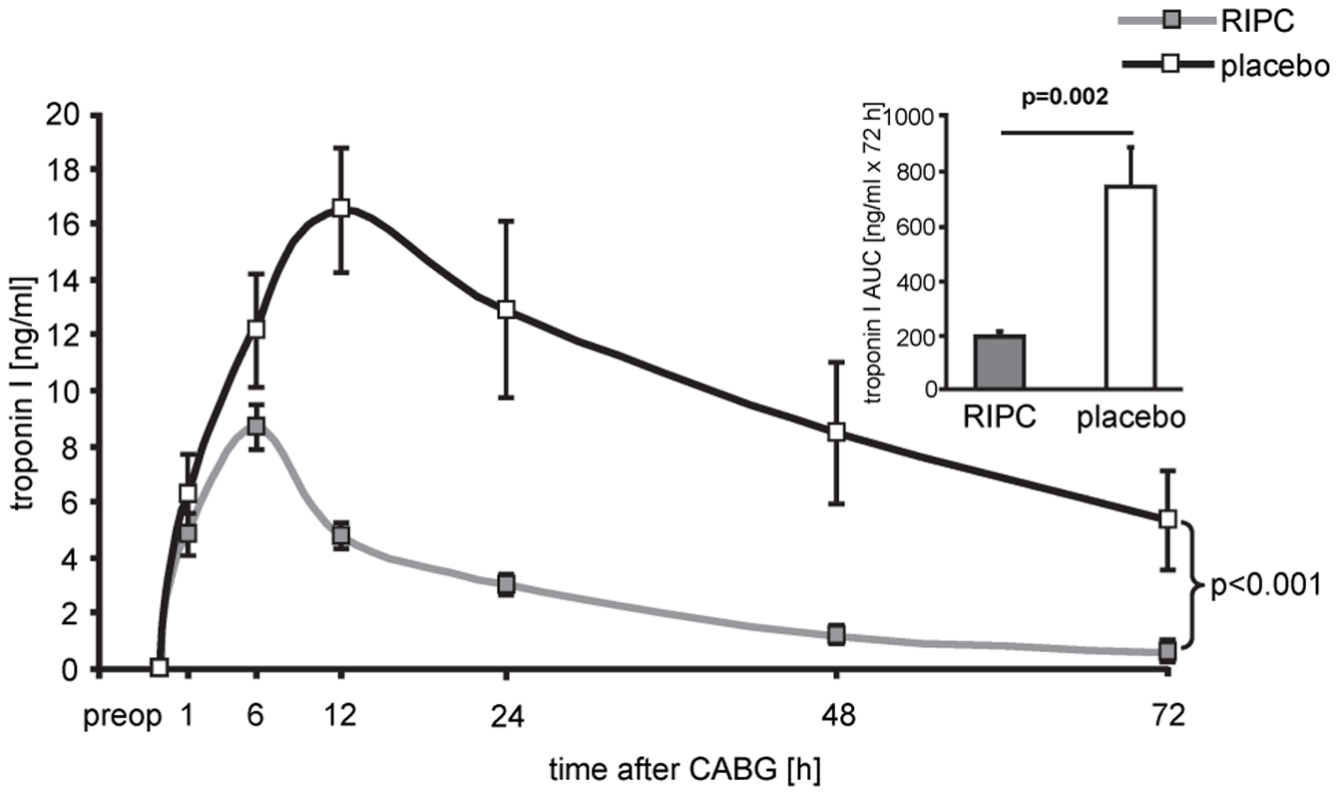
Serum concentration of troponin I. Serum concentration of troponin I before (preop) and over 72 hours after coronary artery bypass grafting (CABG) in patients undergoing remote ischemic preconditioning (RIPC) or not (placebo). Decreased troponin I concentrations confirmed protection by RIPC. Insert: area under the curve (AUC) for serum troponin I concentrations over 72 h.

Demographic data and perioperative characteristics of patients were not different between patients without and with RIPC (Table 1). Preoperative serum cTnI did not differ between patients without and with RIPC. RIPC induced cardioprotection, as evidenced by decreased cTnI (Figure 1) and their AUC over 72 h (194±17 versus 709±129 ng/ml × 72 h, p = 0.002).

**Table 1:** Baseline and perioperative characteristics of patients.

	RIPC (n = 10)	placebo (n = 10)
**Demographics**		
Age (years)	62.6 ± 3.4	65.5 ± 4.2
Sex (male)	9	8
Body weight (kg)	83.2 ± 3.8	84.2 ± 4.0
**Risk factors and comorbidities**		
Diabetes mellitus	0	0
Hypertension	4	9
Hyperlipidemia	3	4
Peripheral vessel disease	2	0
COPD	1	1
Renal disease (creatinine >200 µmol/l)	1	1
**Cardiac status**		
Angina CCS III–IV	3	2
Previous myocardial infarction	0	2
Left-ventricular ejection fraction (%)	51.0 ± 2.4	51.0 ± 2.7
**Medication**		
Aspirin	9	9
Clopidogrel	4	1
β blockers	3	9
Statins	5	9
ACE inhibitors or ARBs	3	5
**Risk scores**		
Additive EuroSCORE	3.8 ± 0.8	4.2 ± 0.5
Logistic EuroSCORE (%)	3.2 ± 0.8	3.2 ± 0.4
EuroSCORE II (%)	0.9 ± 0.1	1.0 ± 0.1
**Intraoperative characteristics**		
Time from end of RIPC or sham to skin incision (min)	5.5 ± 1.0	10.0 ± 1.4
Time from end of RIPC or sham to reperfusion (min)	140.5 ± 8.4	126.8 ± 4.6
Aortic cross-clamp duration (min)	64.0 ± 5.8	70.7 ± 4.3
Cardioplegia (ml)	1428 ± 65	1550 ± 63
Reperfusion time (min)	30.2 ± 4.3	30.9 ± 3.1
Number of bypass grafts	2.7 ± 0.3	2.7 ± 0.2
Number of distal anastomoses	2.9 ± 0.2	3.9 ± 0.4
Transit time graft flow (ml/min)	64.4 ± 12.6	79.5 ± 8.1

Data are mean ± SEM or number. Remote ischemic preconditioning (RIPC), chronic obstructive pulmonary disease (COPD), Canadian Cardiovascular Society score (CCS), angiotensin-converting enzyme (ACE), angiotensin-II-receptor blockers (ARBs), European system for cardiac operative risk evaluation (EuroSCORE).

**Figure 2:**
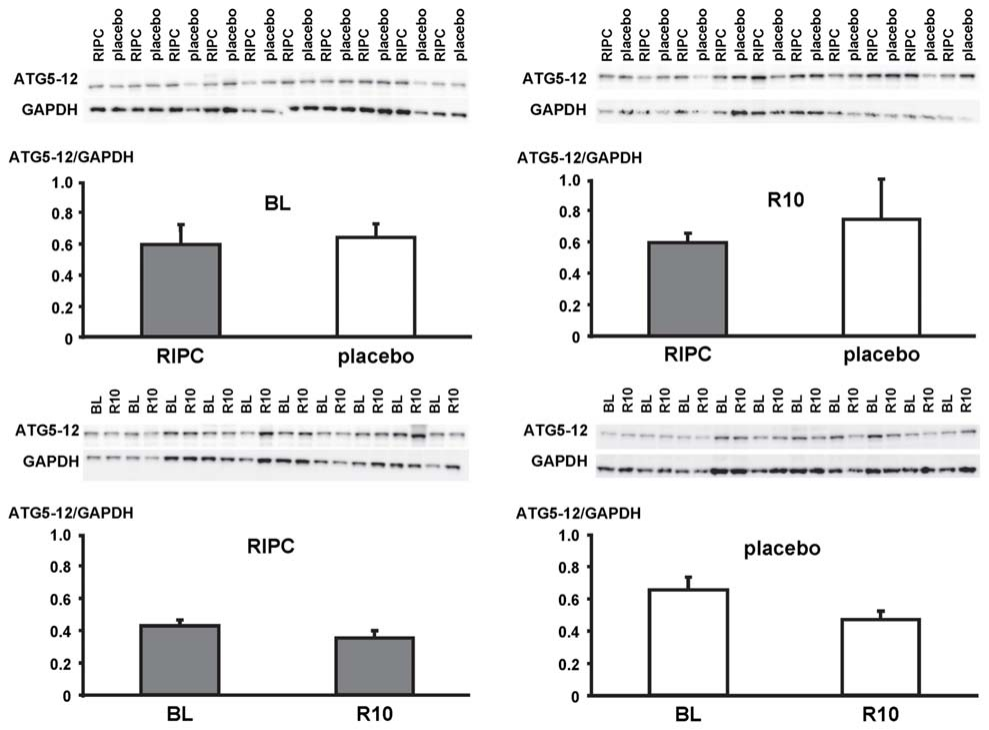
Expression of autophagy-related gene 5–12 complex (ATG5-12). Original Western blots of protein content of ATG5-12 in myocardial biopsies obtained at baseline (BL) before initiation of cardiopulmonary bypass and at 5–10 min reperfusion (R10) from patients undergoing remote ischemic preconditioning (RIPC) or not (placebo). Immunoreactivity of ATG5-12 was normalized to glyceraldehyde 3-phosphate dehydrogenase (GAPDH). Ratios are presented in bar graphs.

**Figure 3:**
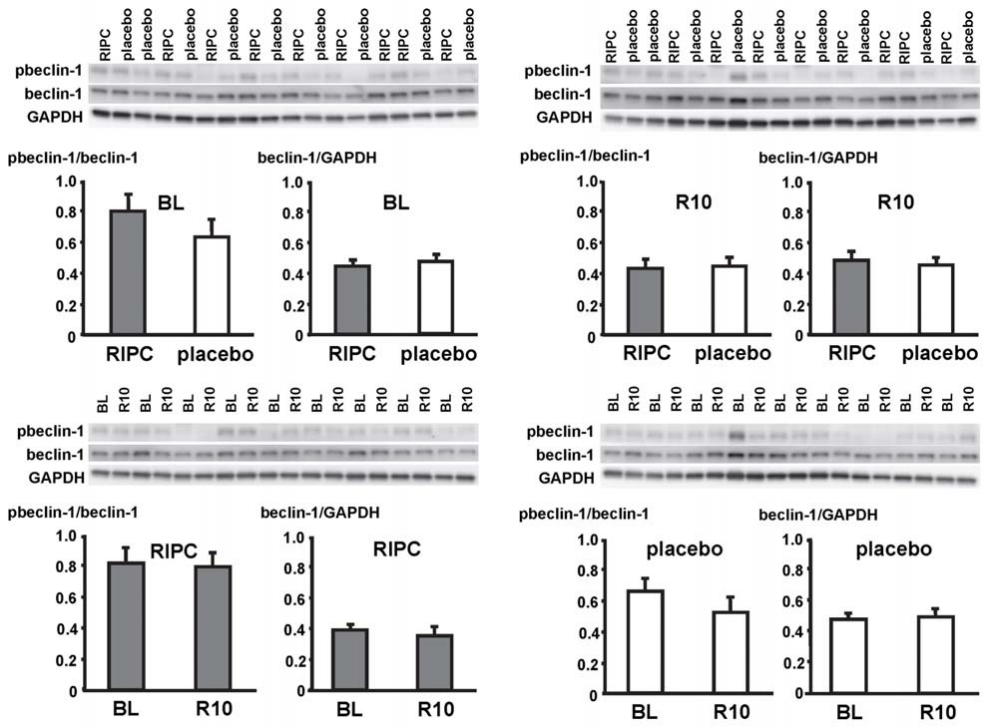
Phosphorylation and expression of beclin-1. Original Western blots of protein content of phosphorylated (p) and total beclin-1 in myocardial biopsies obtained at baseline (BL) before initiation of cardiopulmonary bypass and at 5–10 min reperfusion (R10) from patients undergoing remote ischemic preconditioning (RIPC) or not (placebo). Immunoreactivity of pbeclin-1 was normalized to the respective total protein. Immunoreactivity of total protein was normalized to glyceraldehyde 3-phosphate dehydrogenase (GAPDH). Ratios are presented in bar graphs.

**Figure 4:**
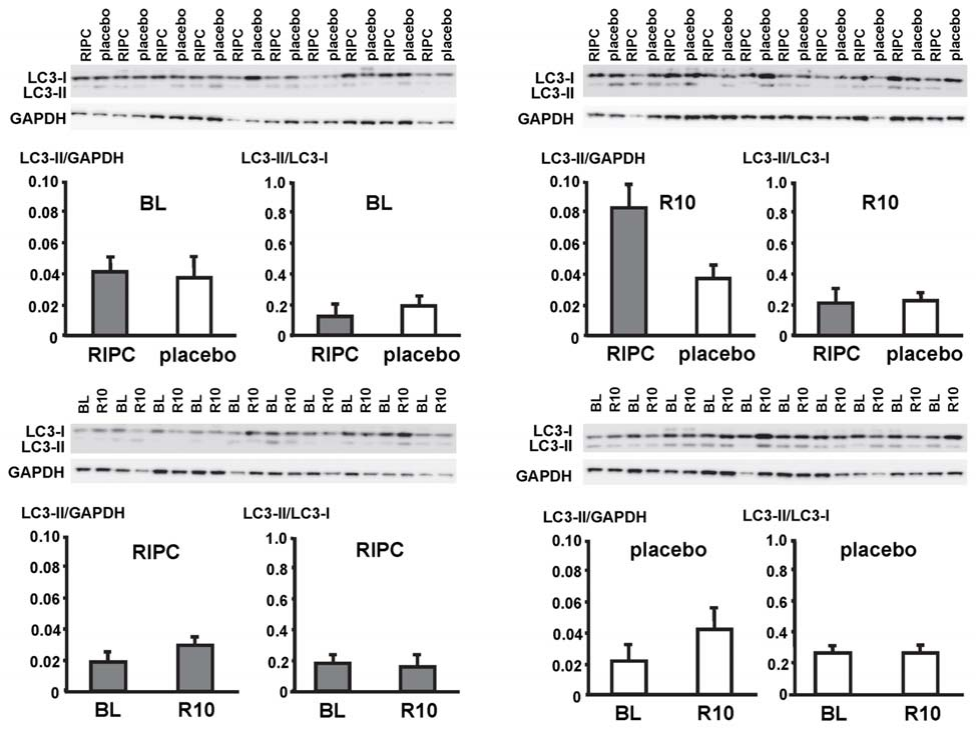
Expression of light chain 3 (LC3). Original Western blots of protein content of LC3 in myocardial biopsies obtained at baseline (BL) before initiation of cardiopulmonary bypass and at 5–10 min reperfusion (R10) from patients undergoing remote ischemic preconditioning (RIPC) or not (placebo). LC3-II (lower band) was normalized to glyceraldehyde 3-phosphate dehydrogenase (GAPDH) and LC3-I (upper band), respectively. Ratios are presented in bar graphs.

**Figure 5:**
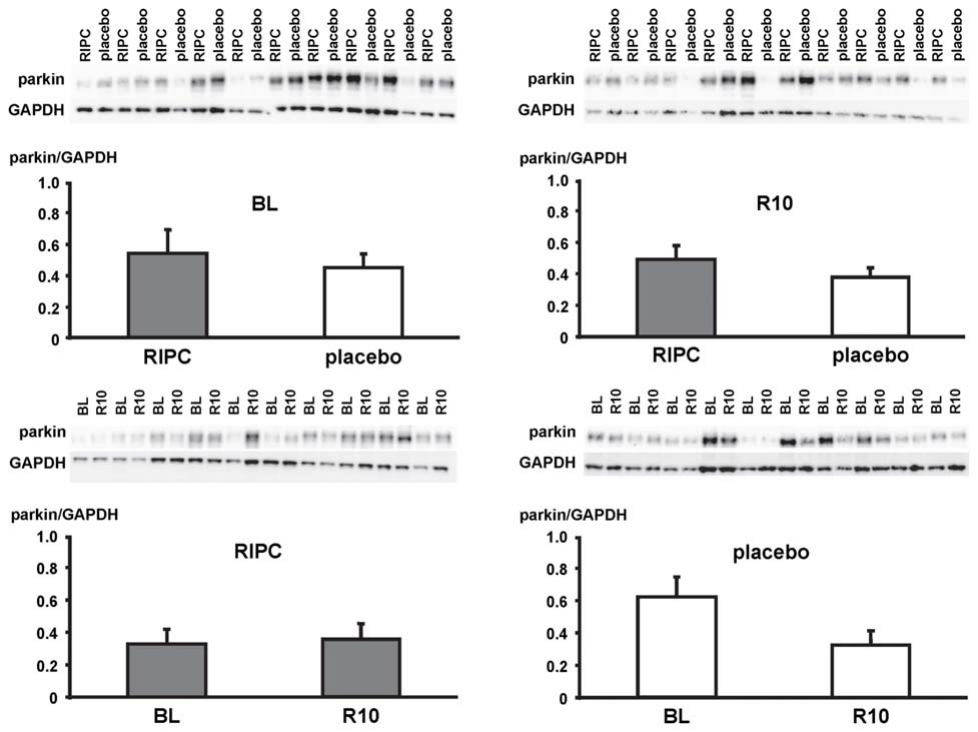
Expression of parkin. Original Western blots of protein content of parkin in myocardial biopsies obtained at baseline (BL) before initiation of cardiopulmonary bypass and at 5–10 min reperfusion (R10) from patients undergoing remote ischemic preconditioning (RIPC) or not (placebo). Immunoreactivity of parkin was normalized to glyceraldehyde 3-phosphate dehydrogenase (GAPDH). Ratios are presented in bar graphs.

**Figure 6:**
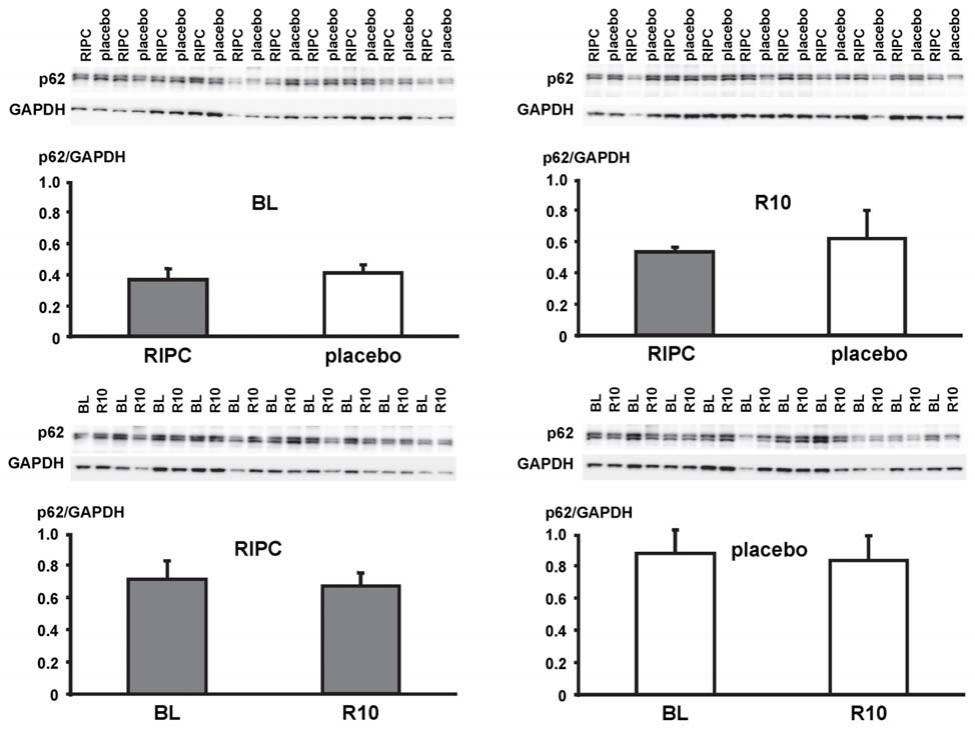
Expression of p62. Original Western blots of protein content of p62 in myocardial biopsies obtained at baseline (BL) before initiation of cardiopulmonary bypass and at 5–10 min reperfusion (R10) from patients undergoing remote ischemic preconditioning (RIPC) or not (placebo). Immunoreactivity of p62 was normalized to glyceraldehyde 3-phosphate dehydrogenase (GAPDH). Ratios are presented in bar graphs.

In LV myocardial biopsies, the expression of ATG5-12, beclin-1, parkin, p62 and the phosphorylation of beclin-1 as well as the formation of LC3-II did not differ between baseline and early reperfusion and between RIPC and placebo, respectively (Figures 2, 3, 4, 5, 6; Table 2).

**Table 2:** Phospho- and total proteins normalized to Ponceau staining.

	BL							R10						
	RIPC			placebo			P-value	RIPC			placebo			P-value
ATG5-12/Ponceau	0.58	±	0.03	0.52	±	0.07	0.517	0.56	±	0.04	0.55	±	0.07	0.950
pbeclin-1/Ponceau	0.34	±	0.04	0.29	±	0.03	0.399	0.22	±	0.04	0.21	±	0.03	0.902
beclin-1/Ponceau	0.44	±	0.04	0.47	±	0.04	0.612	0.49	±	0.04	0.47	±	0.02	0.699
LC3I/Ponceau	0.71	±	0.07	0.82	±	0.16	0.586	0.69	±	0.10	0.87	±	0.16	0.399
LC3II/Ponceau	0.09	±	0.01	0.14	±	0.02	0.103	0.10	±	0.02	0.17	±	0.03	0.154
parkin/Ponceau	0.53	±	0.12	0.45	±	0.14	0.712	0.45	±	0.06	0.38	±	0.10	0.611
p62/Ponceau	0.23	±	0.02	0.23	±	0.01	0.949	0.55	±	0.04	0.60	±	0.05	0.536
pSTAT5/Ponceau	0.59	±	0.13	0.83	±	0.29	0.511	**0.90**	**±**	**0.25**	**0.34**	**±**	**0.06**	**0.062**
STAT5/Ponceau	0.49	±	0.05	0.57	±	0.06	0.398	0.33	±	0.05	0.34	±	0.04	0.925
pSTAT3/Ponceau	0.70	±	0.39	0.88	±	0.45	0.794	**0.51**	**±**	**0.08**	**0.27**	**±**	**0.05**	**0.050**
STAT3/Ponceau	0.31	±	0.02	0.39	±	0.03	0.435	0.54	±	0.04	0.53	±	0.07	0.877
pERK1/2/Ponceau	0.82	±	0.23	0.81	±	0.18	0.981	0.30	±	0.04	0.26	±	0.06	0.650
ERK1/2/Ponceau	0.70	±	0.07	0.83	±	0.10	0.406	0.39	±	0.03	0.43	±	0.04	0.432

	RIPC							Placebo						
	BL			R10			P-value	BL			R10			P-value
ATG5-12/Ponceau	0.47	±	0.03	0.59	±	0.11	0.355	0.66	±	0.10	0.57	±	0.06	0.458
pbeclin-1/Ponceau	0.32	±	0.04	0.30	±	0.05	0.777	0.31	±	0.05	0.34	±	0.04	0.780
beclin-1/Ponceau	0.40	±	0.03	0.37	±	0.02	0.475	0.47	±	0.03	0.49	±	0.03	0.685
LC3I/Ponceau	0.48	±	0.05	0.53	±	0.09	0.510	0.67	±	0.08	0.88	±	0.18	0.207
LC3II/Ponceau	0.09	±	0.02	0.08	±	0.02	0.450	0.14	±	0.02	0.18	±	0.03	0.376
parkin/Ponceau	0.20	±	0.04	0.32	±	0.09	0.270	0.60	±	0.13	0.41	±	0.09	0.103
p62/Ponceau	0.50	±	0.03	0.49	±	0.04	0.881	0.87	±	0.09	0.79	±	0.07	0.340
pSTAT5/Ponceau	0.43	±	0.05	0.74	±	0.18	0.108	0.49	±	0.19	0.31	±	0.08	0.253
STAT5/Ponceau	0.24	±	0.03	0.20	±	0.04	0.101	0.30	±	0.05	0.31	±	0.07	0.828
**pSTAT3/Ponceau**	**0.06**	**±**	**0.02**	**0.49**	**±**	**0.11**	**0.010**	**0.14**	**±**	**0.06**	**0.50**	**±**	**0.09**	**0.081**
STAT3/Ponceau	0.37	±	0.02	0.38	±	0.02	0.827	0.69	±	0.08	0.64	±	0.10	0.763
**pERK1/2/Ponceau**	**0.11**	**±**	**0.02**	**0.54**	**±**	**0.06**	**0.001**	**0.25**	**±**	**0.06**	**0.59**	**±**	**0.08**	**0.020**
ERK1/2/Ponceau	0.38	±	0.03	0.29	±	0.02	0.119	0.62	±	0.06	0.52	±	0.05	0.340

Data are mean ± SEM. Immunoreactivities were compared by paired (within RIPC or placebo, respectively) or unpaired (between RIPC and placebo) Student t-tests. Protein content of autophagy-related gene 5–12 complex (ATG5-12), phosphorylated (p) and total beclin-1, light chain 3 (LC3), parkin, p62, signal transducer and activator of transcription 5 (STAT5), STAT3, and extracellular signal-regulated protein kinase 1/2 (ERK1/2) in myocardial biopsies obtained at baseline (BL) before initiation of cardiopulmonary bypass and at 5–10 min reperfusion (R10) from patients undergoing remote ischemic preconditioning (RIPC) or not (placebo).

**Figure 7:**
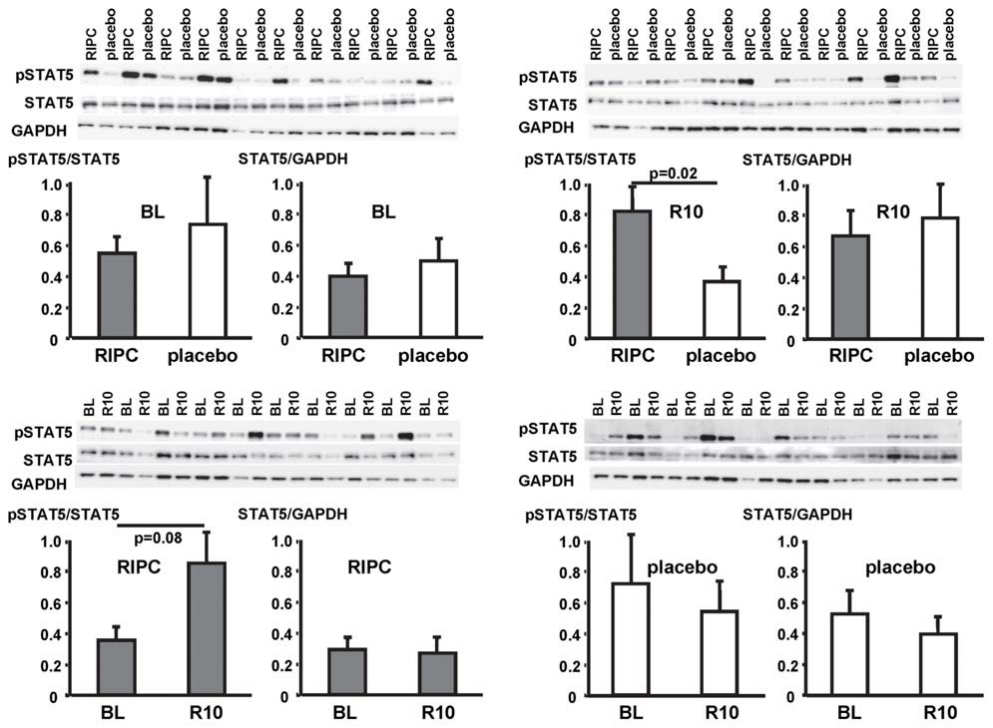
Activation of signal transducer and activator of transcription 5 (STAT5) by ischemia/reperfusion. Original Western blots of phosphorylated (p) and total STAT5 content in myocardial biopsies obtained at baseline (BL) before initiation of cardiopulmonary bypass and at 5–10 min reperfusion (R10) from patients undergoing remote ischemic preconditioning (RIPC) or not (placebo). Immunoreactivitiy of pSTAT5 was normalized to the respective total protein. Ratios are presented in bar graphs.

**Figure 8:**
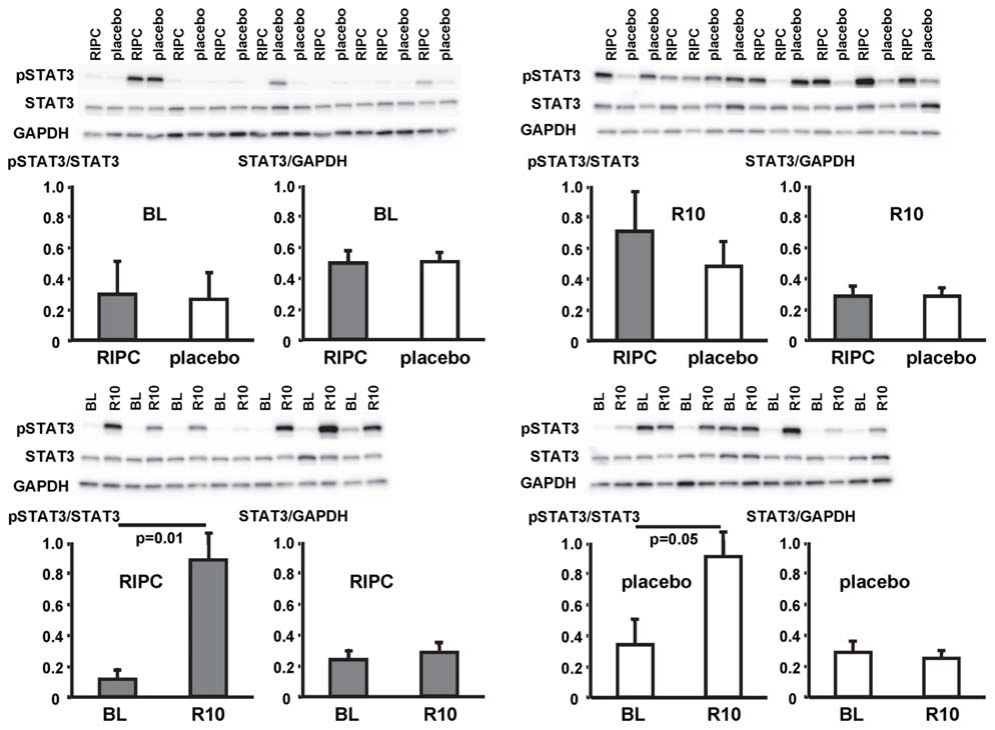
Activation of signal transducer and activator of transcription 3 (STAT3) by ischemia/reperfusion. Original Western blots of phosphorylated (p) and total STAT3 content in myocardial biopsies obtained at baseline (BL) before initiation of cardiopulmonary bypass and at 5–10 min reperfusion (R10) from patients undergoing remote ischemic preconditioning (RIPC) or not (placebo). Immunoreactivitiy of pSTAT3 was normalized to the respective total protein. Ratios are presented in bar graphs.

**Figure 9:**
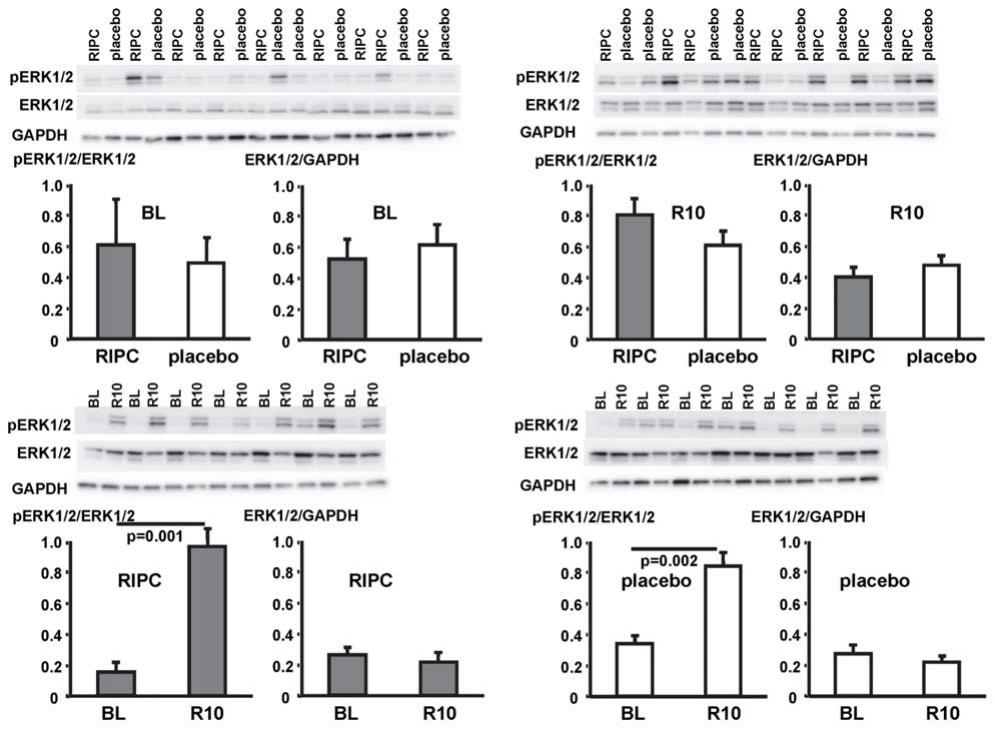
Activation of signal transducer and activator of extracellular signal-regulated protein kinase 1/2 (ERK1/2) by ischemia/reperfusion. Original Western blots of phosphorylated (p) and total ERK1/2 content in myocardial biopsies obtained at baseline (BL) before initiation of cardiopulmonary bypass and at 5–10 min reperfusion (R10) from patients undergoing remote ischemic preconditioning (RIPC) or not (placebo). Immunoreactivitiy of pERK1/2 was normalized to the respective total protein. Ratios are presented in bar graphs.

In contrast, STAT5-phosphorylation was greater at early reperfusion only in patients with RIPC. The phosphorylation of STAT3 and ERK1/2 was greater at early reperfusion both with RIPC and placebo (Figures 7, 8, 9; Table 2).

## Discussion

We did not detect differences in the abundance of key autophagy proteins in LV myocardium of patients undergoing CABG, not with ischemia/reperfusion per se and not between placebo and RIPC. However, we confirmed protection by RIPC with reduction in serum cTnI [4] and also confirmed the increased phosphorylation of STAT3 and ERK1/2 at early reperfusion and the increased phosphorylation of STAT5 with RIPC over placebo [6]. The difference in cTnI between RIPC in placebo was somewhat larger in the present study with only 10 patients per group than in the main trial, and the difference in STAT 5 was somewhat less pronounced than previously reported, both probably through biological variability at small sample size.

Autophagy in our LV biopsy samples was assessed by established markers [12]: beclin-1 catalyzes the modification of lipids to form the initial autophagosomal membrane [20] and is phosphorylated by DAP-kinase [21]. The conjugation of ATG12 to ATG5 is essential for the extension of the membrane, and LC3 (or ATG8) is processed from the soluble form (LC3-I) to LC3-II, which is then associated with the autophagosome until it is degraded in the lysosome [20]. The adaptor protein p62 binds to organelles or proteins which are ubiquitinated and facilitates their enclosure by the autophagosome. Parkin is an ubiquitin ligase, which translocates to damaged mitochondria to ubiquitinate mitochondrial proteins for their autophagic removal [22].

We sampled LV biopsies only at baseline before initiation of CPB and at 5–10 min reperfusion after aortic unclamping and, therefore, cannot exclude autophagy activation at earlier/later time points. In isolated rat hearts, the expression of LC3-II and beclin-1 was enhanced immediately after repeated cycles of ischemia and reperfusion to induce ischemic preconditioning [16]. In pigs, chloramphenicol enhanced the expression of LC3-II and beclin-1 after 10 min and reduced infarct size [23]. In contrast to our data, Jahania et al. have reported decreased expression of ATG5-12, beclin-1, LC3-I, LC3-II, and p62 in biopsies from right atrial appendages of CABG or valve surgery patients after removal of the aortic cross-clamp and weaning from CPB [13]. In our study, biopsies were taken from the LV rather than right atrial appendage, and the time interval between the baseline sample and the sample after CPB was twice as long (145±35 [13] versus 67±16 min). When the autophagy process is advanced, autophagic proteins are also degraded [24], [25], potentially explaining the depletion of autophagy proteins during longer CPB. Supporting such notion, transcript and protein levels of autophagic proteins (LC3-II, beclin-1, and ATG5-12) were also decreased in failing LV myocardium of patients with idiopathic dilated cardiomyopathy after explantation of a LV assist device [11].

There are a number of limitations of our current analysis. First, we analyzed biopsies from only a small number of patients. Due to the small number and size of biopsy samples we can not exclude false negative findings. However, we could confirm cardioprotection and STAT5 activation by RIPC in this cohort. Second, we analyzed biopsies at only two time points, i.e., at baseline before and after about 1 h ischemia at early reperfusion. Accordingly, we may have missed an increase in the abundance of autophagic proteins at earlier time points and/or their decrease with a longer duration of ischemia and/or reperfusion. Thus, we could not exclude that with multiple biopsy sampling we might have obtained positive results. However, multiple sampling was not possible for ethical reasons. Third, we analyzed total myocardial proteins and cannot allocate the proteins to different cellular and subcellular compartments. In particular, parkin mediates cardioprotection by ischemic preconditioning through p62 and mitophagy [17], but we were unable to look at mitochondrial proteins. Again, for ethical reasons, sufficient tissue sampling for immunoblotting on isolated mitochondria was not possible.

Nevertheless, under the given circumstances of our study we saw protection by RIPC but no apparent change in autophagic proteins.

Accordingly, these autophagic proteins appear to be no prerequisite for the observed cardioprotection.

## Supporting Information

Figure S1 **Original Ponceau stainings with the respective glyceraldehyde 3-phosphate dehydrogenase (GAPDH) immunreactivities.** Western blots were used to detect expression of autophagy-related gene 5-12 complex (ATG5-12) (immunreactivities in Figure 2) and parkin (immunreactivities in Figure 5) in myocardial biopsies obtained at baseline (BL) before initiation of cardiopulmonary bypass and at 5-10 min reperfusion (R10) from patients undergoing remote ischemic preconditioning (RIPC) or not (placebo). GAPDH/Ponceau-ratios were presented numerically.
(TIF)

Figure S2 **Original Ponceau stainings with the respective glyceraldehyde 3-phosphate dehydrogenase (GAPDH) immunreactivities.** Western blots were used to detect phosphorylated and total beclin-1 (immunreactivities in Figure 3) in myocardial biopsies obtained at baseline (BL) before initiation of cardiopulmonary bypass and at 5–10 min reperfusion (R10) from patients undergoing remote ischemic preconditioning (RIPC) or not (placebo). GAPDH/Ponceau-ratios were presented numerically.
(TIF)

Figure S3 **Original Ponceau stainings with the respective glyceraldehyde 3-phosphate dehydrogenase (GAPDH) immunreactivities.** Western blots were used to detect phosphorylated and total signal transducer and activator of transcription 3 (STAT5) (immunreactivities in Figure 7) and expression of p62 (immunreactivities in Figure 6) and light chain 3 (LC3) (immunreactivities in Figure 4) in myocardial biopsies obtained at baseline (BL) before initiation of cardiopulmonary bypass and at 5–10 min reperfusion (R10) from patients undergoing remote ischemic preconditioning (RIPC) or not (placebo). GAPDH/Ponceau-ratios were presented numerically.
(TIF)

Figure S4 **Original Ponceau stainings with the respective glyceraldehyde 3-phosphate dehydrogenase (GAPDH) immunreactivities.** Western blots were used to detect phosphorylated and total signal transducer and activator of transcription 3 (STAT3) (immunreactivities in Figure 8) and extracellular signal-regulated protein kinase 1/2 (ERK1/2) (immunreactivities in Figure 9) in myocardial biopsies obtained at baseline (BL) before initiation of cardiopulmonary bypass and at 5–10 min reperfusion (R10) from patients undergoing remote ischemic preconditioning (RIPC) or not (placebo). GAPDH/Ponceau-ratios were presented numerically.
(TIF)
