# Correction: No Evidence for Activated Autophagy in Left Ventricular Myocardium at Early Reperfusion with Protection by Remote Ischemic Preconditioning in Patients Undergoing Coronary Artery Bypass Grafting

**DOI:** 10.1371/journal.pone.0105663

**Published:** 2014-08-07

**Authors:** The PLOS ONE Staff

There is an error in the name of the funding organization, Hans und Gerti Fischer Foundation. Please refer to the correct funding statement below:

This research was supported by the Dr. Heinz-Horst Deichmann Foundation and the Hans und Gertie Fischer Foundation. The funders had no role in study design, data collection and analysis, decision to publish, or preparation of the manuscript.
