# Supplementary figures and images for: No Evidence for Activated Autophagy in Left Ventricular Myocardium at Early Reperfusion with Protection by Remote Ischemic Preconditioning in Patients Undergoing Coronary Artery Bypass Grafting
Source: PLoS One. 2014 May 5;9(5):e96567. doi: 10.1371/journal.pone.0096567 (PMC4010496; doi:10.1371/journal.pone.0096567)

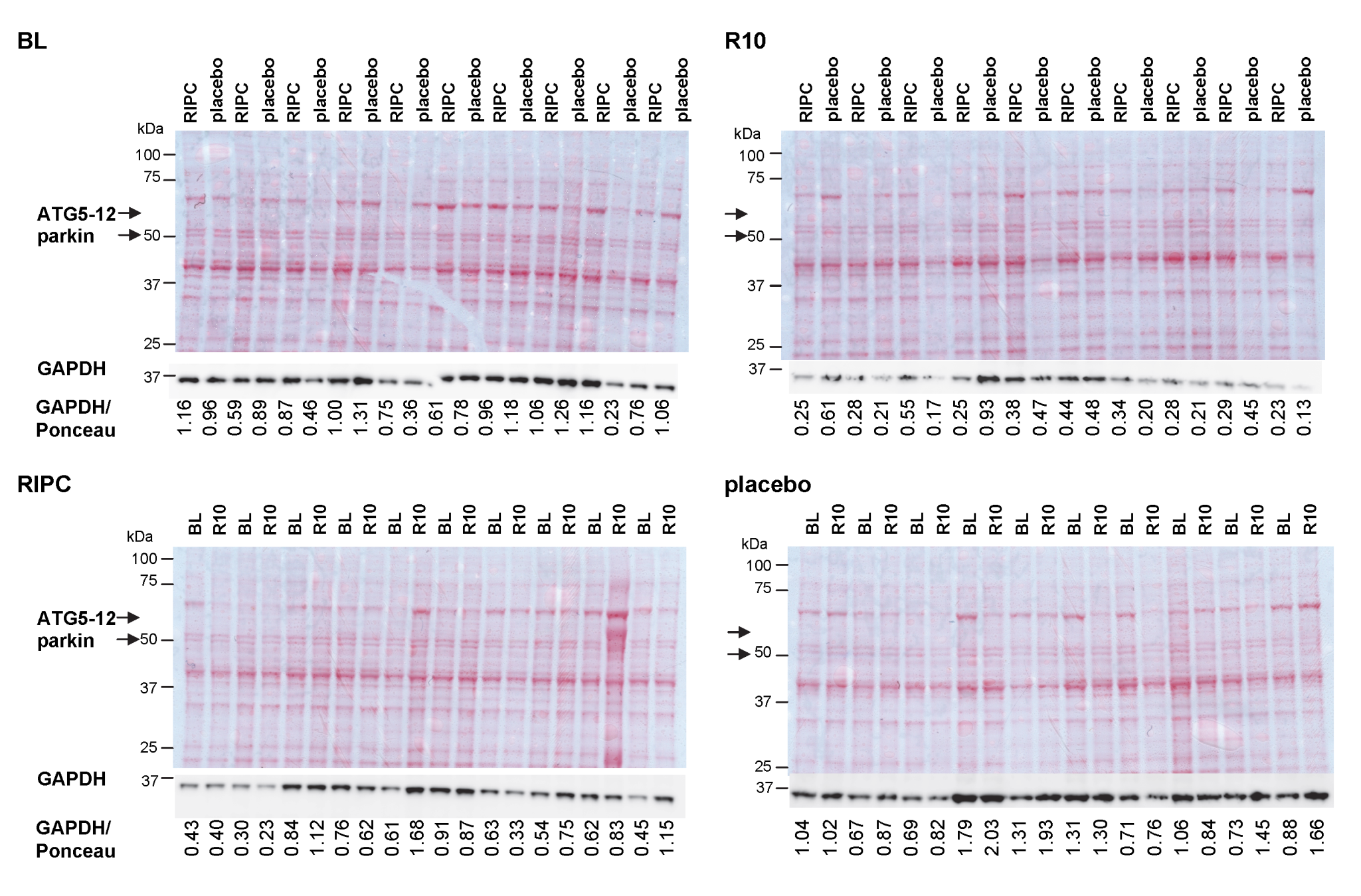

Supplement: Figure S1 — Original Ponceau stainings with the respective glyceraldehyde 3-phosphate dehydrogenase (GAPDH) immunreactivities. Western blots were used to detect expression of autophagy-related gene 5-12 complex (ATG5-12) (immunreactivities in Figure 2) and parkin (immunreactivities in Figure 5) in myocardial biopsies obtained at baseline (BL) before initiation of cardiopulmonary bypass and at 5-10 min reperfusion (R10) from patients undergoing remote ischemic preconditioning (RIPC) or not (placebo). GAPDH/Ponceau-ratios were presented numerically. (TIF) [file pone.0096567.s001.tif]

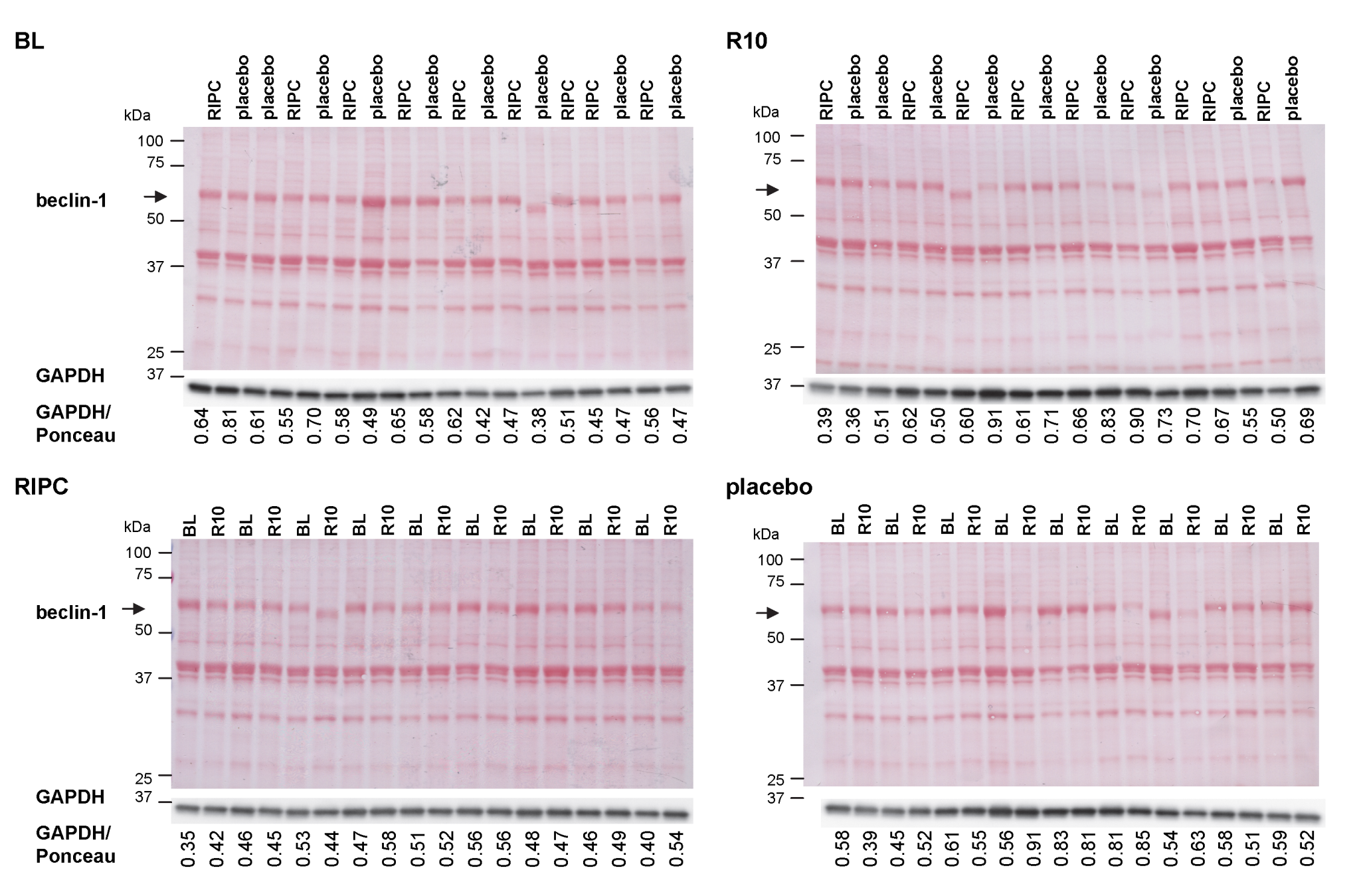

Supplement: Figure S2 — Original Ponceau stainings with the respective glyceraldehyde 3-phosphate dehydrogenase (GAPDH) immunreactivities. Western blots were used to detect phosphorylated and total beclin-1 (immunreactivities in Figure 3) in myocardial biopsies obtained at baseline (BL) before initiation of cardiopulmonary bypass and at 5–10 min reperfusion (R10) from patients undergoing remote ischemic preconditioning (RIPC) or not (placebo). GAPDH/Ponceau-ratios were presented numerically. (TIF) [file pone.0096567.s002.tif]

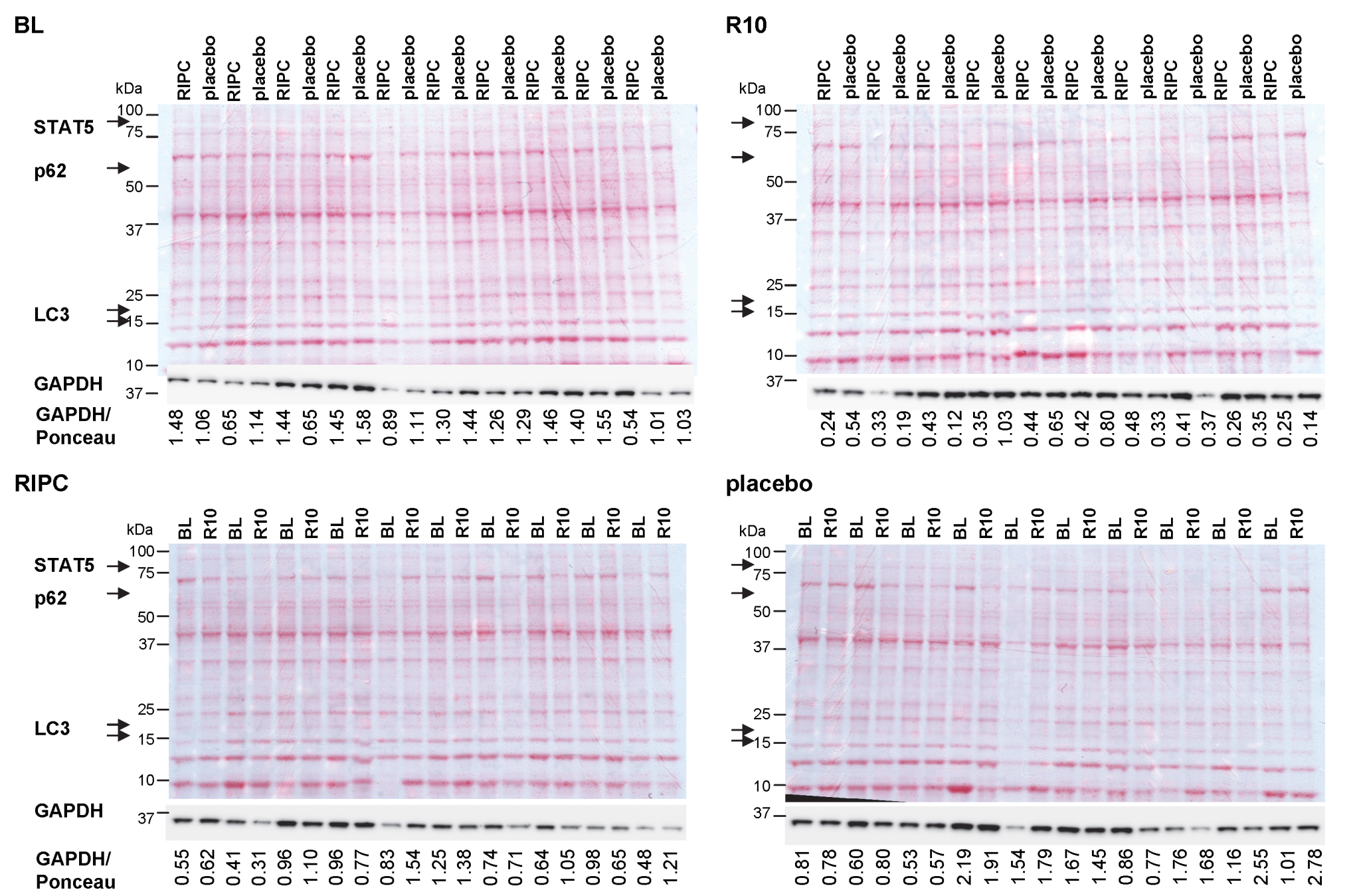

Supplement: Figure S3 — Original Ponceau stainings with the respective glyceraldehyde 3-phosphate dehydrogenase (GAPDH) immunreactivities. Western blots were used to detect phosphorylated and total signal transducer and activator of transcription 3 (STAT5) (immunreactivities in Figure 7) and expression of p62 (immunreactivities in Figure 6) and light chain 3 (LC3) (immunreactivities in Figure 4) in myocardial biopsies obtained at baseline (BL) before initiation of cardiopulmonary bypass and at 5–10 min reperfusion (R10) from patients undergoing remote ischemic preconditioning (RIPC) or not (placebo). GAPDH/Ponceau-ratios were presented numerically. (TIF) [file pone.0096567.s003.tif]

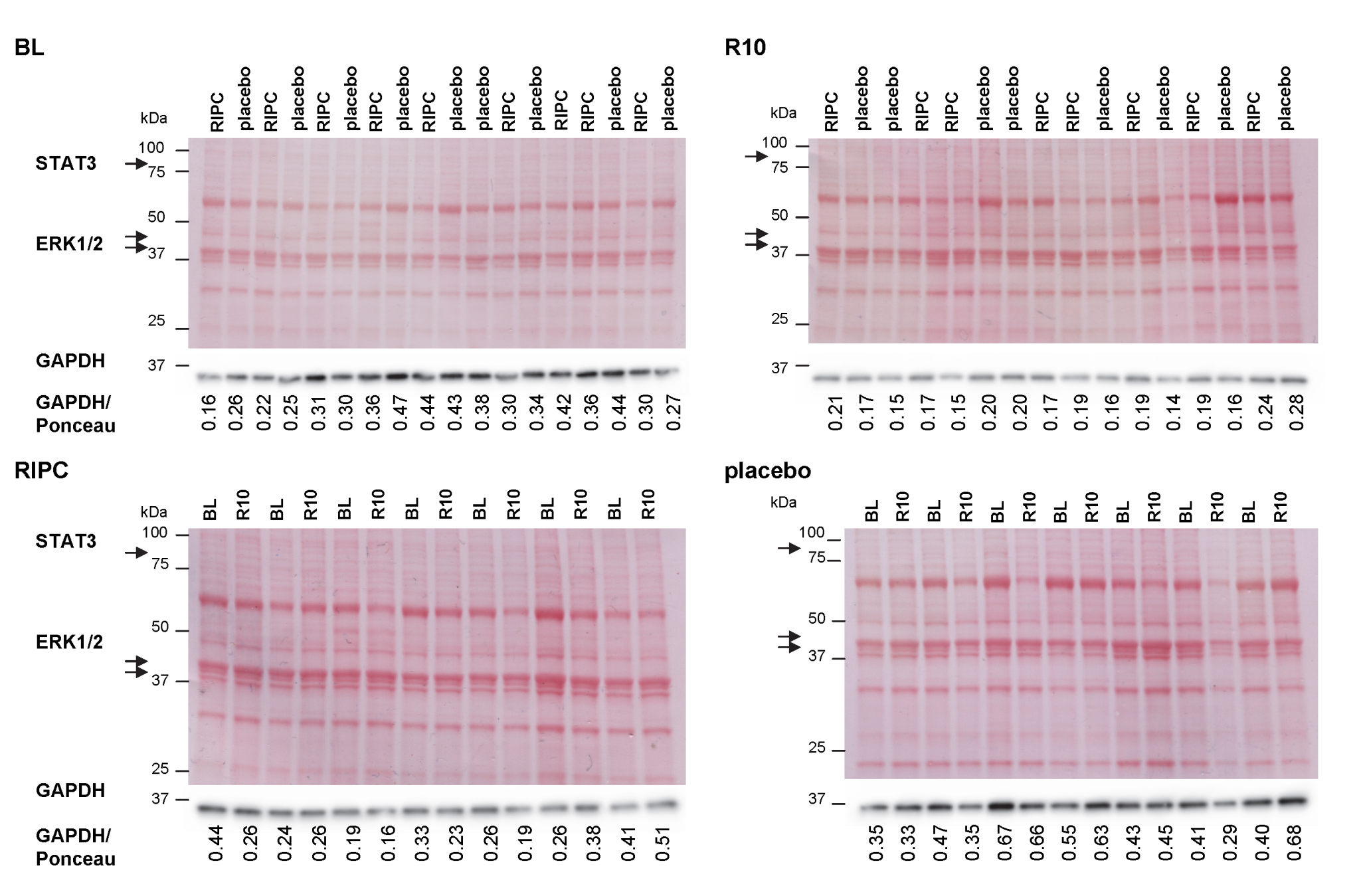

Supplement: Figure S4 — Original Ponceau stainings with the respective glyceraldehyde 3-phosphate dehydrogenase (GAPDH) immunreactivities. Western blots were used to detect phosphorylated and total signal transducer and activator of transcription 3 (STAT3) (immunreactivities in Figure 8) and extracellular signal-regulated protein kinase 1/2 (ERK1/2) (immunreactivities in Figure 9) in myocardial biopsies obtained at baseline (BL) before initiation of cardiopulmonary bypass and at 5–10 min reperfusion (R10) from patients undergoing remote ischemic preconditioning (RIPC) or not (placebo). GAPDH/Ponceau-ratios were presented numerically. (TIF) [file pone.0096567.s004.tif]
